# Female-specific alterations in insulin dynamics are associated with glucose tolerance in a Glut1DS mouse model

**DOI:** 10.1242/dmm.052836

**Published:** 2026-09-21

**Authors:** Tamio Furuse, Tomoko Kushida, Akiko Shinogi, Mao Ozaki, Ryoko Yanagisawa, Masaru Tamura

**Affiliations:** ^1^ Mouse Phenomics Division, RIKEN BioResource Research Center, 3-1-1 Koyadai, Tsukuba, Ibaraki 3050074, Japan

**Keywords:** Glut1DS, Mouse model, SLC2A1, Glucose metabolism, Insulin secretion

## Abstract

Glucose transporter type 1 deficiency syndrome (Glut1DS) is a rare autosomal dominant genetic disorder caused by mutations in *SLC2A1*, which results in impaired glucose transport across the blood−brain barrier. Patients with Glut1DS present with various symptoms, including seizures, delayed development and movement disorders. The current first-line therapy − ketogenic diet − has shown variable efficacy, highlighting the need to explore alternative therapeutic strategies. This study aimed to investigate comprehensive phenotypic characteristics of the Glut1DS mouse model B6(D2)-Slc2a1<Rgsc200>/1Rbrc to establish foundational data for identifying novel therapeutic targets. The *Slc2a1^Rgsc200^* mutant mouse was phenotypically characterized at the Japan Mouse Clinic of the RIKEN BioResource Research Center by using a phenotyping pipeline that included intraperitoneal glucose tolerance test (IPGTT) and clinical blood chemistry analyses. Heterozygous Glut1DS mutants exhibited no differences in basal plasma glucose or glycosylated hemoglobin A1c levels when compared to wild-type controls. However, female heterozygous mutants showed decreased glucose levels during IPGTT, accompanied by increased plasma insulin levels following glucose administration. These findings support recent evidence linking insulin signaling to seizure control in patients with Glut1DS, as demonstrated in human studies.

## Teaser

**Summary:** Comprehensive phenotyping of a glucose transporter type 1 deficiency syndrome mouse model reveals sex-specific changes in insulin response and glucose tolerance, supporting clinical strategies targeting insulin signaling.

## INTRODUCTION

Glucose transporter type 1 deficiency syndrome (Glut1DS; OMIM #606777) is a rare autosomal dominant genetic disorder caused by a deficiency of solute carrier family 2 member 1 [SLC2A1; also known as glucose transporter type 1 (GLUT1)], encoded by the *SLC2A1* gene (Vannucci et al., 1997; Seidner et al., 1998; Barros et al., 2007). SLC2A1 plays a critical role in the transport of glucose, the primary energy source for the brain, across the blood−brain barrier. Deficiency of the SLC1A1 protein results in an insufficient glucose supply to the brain, leading to a spectrum of neurological symptoms, including seizures, delayed development, intellectual disability, movement disorders, and hypotonia (Barros et al., 2007; De Giorgis et al., 2015; Leen et al., 2010; Olivotto et al., 2022; Vannucci et al., 1997). The diagnosis of Glut1DS is based on a combination of clinical features, including hypoglycorrhachia [i.e. low levels of glucose in the cerebrospinal fluid (CSF)], and genetic testing to identify mutations in SLC2A1 (Klepper et al., 2020). To date, no pharmacological treatment has been proven effective for Glut1DS. The current first-line therapy is a ketogenic diet (KD). By restricting carbohydrate intake, KD induces a state of ketosis, enabling patients to utilize ketone bodies, such as β-hydroxybutyrate and acetoacetate, as alternative energy sources for the brain. These ketone bodies cross the blood−brain barrier via the monocarboxylate transporter 1 [known as solute carrier family 16 member 1 (SLC16A1)] (Tang et al., 2019; Yang et al., 2019). Early administration of KD has been effective in controlling epileptic seizures, movement disorders and cognitive impairments associated with Glut1DS (Tang et al., 2019). However, KD therapy is not universally effective and may provide inadequate or no therapeutic benefits to some patient populations (Bekker et al., 2019; Hao et al., 2017). Therefore, alternative therapeutic strategies are required. Although Glut1DS is caused by impaired glucose transport to the brain, alterations in systemic glucose metabolism in patients and animal models remain largely unexplored.

Since human studies are complicated by the heterogeneity of pathogenesis and symptoms due to genetic and environmental variability, mouse models of *Slc2a1* mutations provide an invaluable platform for preclinical research, as their uniform genetic background and controlled environmental conditions have enabled more precise investigations (Furuse et al., 2019; Meyer et al., 2018; Nakamura et al., 2017; Tang et al., 2017; Wang et al., 2006). This study focuses on comprehensive phenotyping, including assessments of glucose metabolism and insulin release in a missense mutant mouse model of *Slc2a1*, known as B6(D2)-Slc2a1<Rgsc200>/1Rbrc (Furuse et al., 2019). This mutant strain carries the *Slc2a1^Rgsc200^* allele (formerly known as *Glut1^Rgsc200^*) induced by N-ethyl-N-nitrosourea (ENU). This mutation results in a missense alteration in the *Slc2a1* gene, leading to a Ser324Pro substitution in SLC2A1 (Furuse et al., 2019). Previous studies have shown that heterozygous *Slc2a1^Rgsc200^* mutant mice exhibit several phenotypes associated with Glut1DS, including seizure-like behavior, embryonic lethality in homozygotes, decreased levels of glucose in the cerebrospinal fluid, contextual learning deficits, reduced body size, abnormal electroencephalogram (EEG) patterns, longer wake durations and shorter non-rapid eye movement (NREM) sleep durations (Furuse et al., 2019). Moreover, these mutants exhibited impaired glucose transport and elevated brain glucose utilization (Furuse et al., 2019).

Phenotyping in mouse models often focuses on specific traits based on the researchers' interests, leading to potential biases and overlooked phenotypes. To address this issue, systematic and comprehensive phenotyping pipelines have been developed, and are widely used in biological and medical studies (Gates et al., 2011; Hrabě De Angelis et al., 2015; Meehan et al., 2017; Rozman et al., 2018). At the Japan Mouse Clinic (JMC) of the RIKEN BioResource Research Center (BRC), we have developed phenotyping pipelines 1 and 2 (PL1 and PL2), which have contributed valuable findings (Hirata et al., 2022; Matsumura et al., 2020; Uemura et al., 2022). The aim of this study was to screen for previously unknown functional changes in *Slc2a1^Rgsc200^* mice. The results of this study suggest potential clinical phenotypes in patients with Glut1DS − including abnormalities in glucose metabolism − and could help identify new therapeutic targets for Glut1DS by utilizing PL1 within our comprehensive mouse phenotyping platform.

## RESULTS

### Female heterozygous mutants exhibited altered glucose tolerance evaluated with intraperitoneal glucose tolerance test

In the clinical blood chemistry test conducted with 11-week-old mice as a part of PL1, heterozygous mutant and wild-type mice showed no significant differences in plasma glucose levels or percentage of glycosylated hemoglobin A1c (HbA1c) based on a two-way ANOVA when evaluating the effects of genotype and sex (Fig. 1A,B). However, the intraperitoneal glucose tolerance test (IPGTT) performed at 14 weeks of age as part of PL1, female heterozygous mutants exhibited significantly altered blood glucose levels following intraperitoneal (i.p.) glucose administration compared with wild-type counterparts, as revealed by a two-way ANOVA (effects of genotype) (Fig. 2A,B). Šidák's multiple comparisons post-hoc test following ANOVA showed that blood glucose levels were significantly lower in heterozygous mutants (Fig. 2A). Additionally, two-way ANOVA (effects of genotype and sex) of the area under the curve (AUC), an indicator of glucose tolerance calculated from Fig. 2A,B, revealed a significant interaction between genotype and the main effect of genotype (Fig. 2C). In the post-hoc analysis, the AUC was significantly reduced only in female heterozygous mutants (Fig. 2C) (Šidák's multiple comparisons test).

**Fig. 1.**
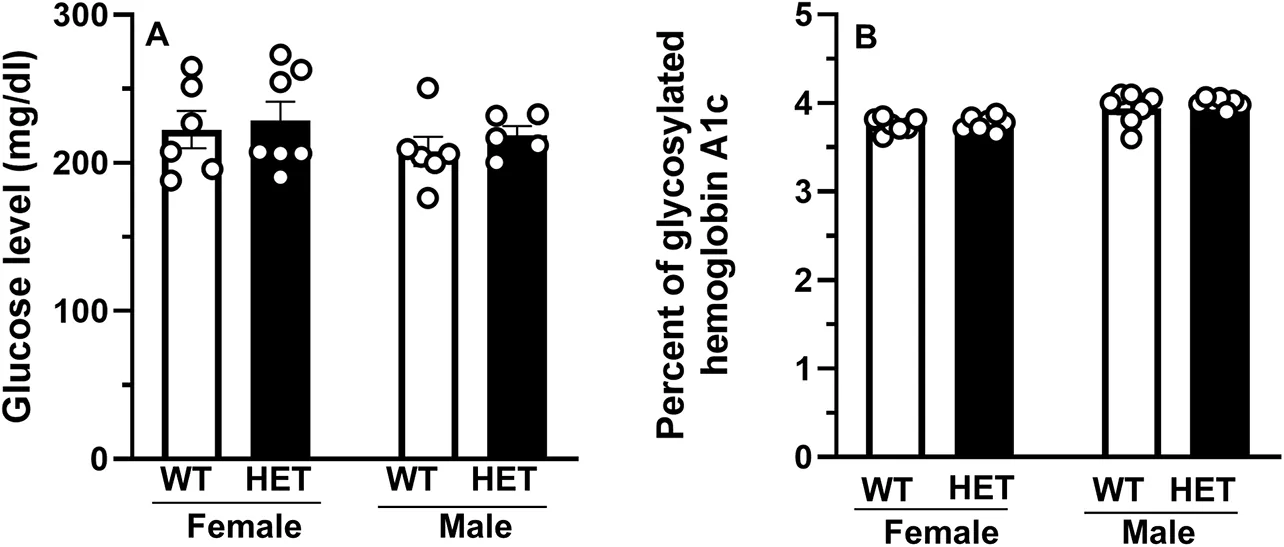
**Glucose-related features measured in clinical blood chemistry test.** (A) Bar graphs represent the mean plasma glucose levels for female and male wild-type (WT) and heterozygous (HET) mice. Two-way ANOVA: interaction between genotype and sex, *F_1, 20_*=0.04353, *P*=0.8368; effect of genotype: *F_1, 20_*=0.5824, *P*=0.4543; effect of sex: *F_1, 20_*=1.194, *P*=0.2876, with *F* denoting the *F*-statistic in ANOVA, and _*1*_ and _*20*_ the numerator and denominator degree of freedom, respectively. Sample sizes: WT females, *n=*6; WT males, *n=*6; HET females *n=*7; HET males, *n=*5. Plasma samples that did not pass the quality control threshold (see Materials and Methods) were excluded from the analyses. (B) Bar graphs represent the mean level of glycosylated hemoglobin (HbA1c). Two-way ANOVA: interaction between genotype and sex, *F_1, 24_*=0.3744, *P*=0.5464; effect of genotype: *F_1, 24_*=0.7812, *P*=0.3855; effect of sex: *F_1, 24_*=24.97, *P*<0.0001). Sample sizes: *n=*7 per group. All error bars indicate the ±standard error of the mean (±s.e.m.); circles indicate individual values.

**Fig. 2.**
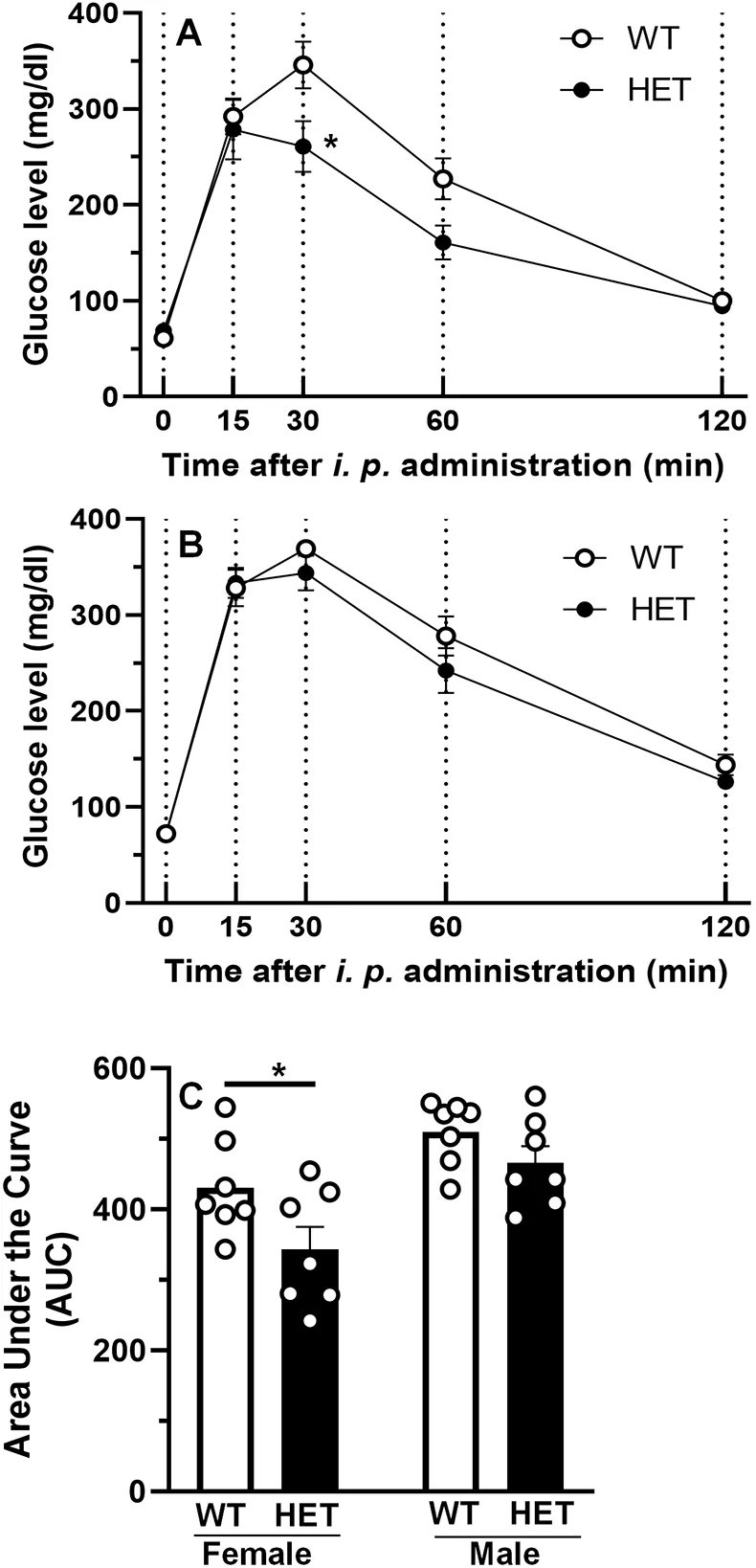
**Results of intraperitoneal glucose tolerance test (IPGTT).** (A,B) Time-course of blood glucose level changes in female (A) and male (B) wild-type (WT) and heterozygous (HET) mice. Plotted are the mean blood glucose level for each group at each time point. (A) Two-way ANOVA: interaction between genotype and time, *F_4, 60_*=2.327, with *F* denoting the *F*-statistic in ANOVA, and _*4*_ and _*60*_ the numerator and denominator degree of freedom, respectively. *P*=0.0664; effect of genotype: *F_1, 60_*=7.488, *P*=0.0082; effect of time: *F*_*4*, *60*,_=64.97, *P*<0.0001. Šidák's multiple-comparisons test [WT vs HET mice]: 0 min, adjusted *P=*0.9994; 15 min, adjusted *P=*0.9912; 30 min, adjusted *P=*0.0111; 60 min, adjusted *P=*0.0757; 120 min, adjusted *P=*0.9999. (B) Two-way ANOVA: interaction between genotype and time, *F_4, 60_*=0.6957, *P*=0.5979; effect of genotype: *F_1_*_,_ *_60_*=2.509, *P*=0.1184; effect of time: *F*_*4*, *60*,_=142.7, *P*<0.0001. (C) Bar graphs represent the mean area under the curve (AUC) calculated using the time-course curve of blood glucose levels shown in A and B for each group. Two-way ANOVA, interaction between genotype and sex, *F_1, 24_*=0.7412, *P*=0.3978; effect of genotype: *F_1, 24_*=6.816, *P*=0.0153; effect of sex: *F_1, 24_*=16.14, *P=*0.0005, Šidák's multiple-comparisons test, WT vs HET in females, adjusted *P*=0.0430; WT vs HET in males, adjusted *P*=0.4039. All error bars indicate the ±standard error of the mean (±s.e.m.); individual values are indicated by circles. Sample sizes, *n=*7 per group. *, adjusted *P*<0.05 in Šidák's multiple-comparisons test.

### Plasma insulin levels after i.p. injection of glucose increased only in heterozygous females

To further investigate the alterations in glucose tolerance observed in heterozygous mutants by IPGTT, we generated an additional cohort of mice (independent of that used with the phenotyping pipeline) and measured plasma insulin levels following i.p. saline or glucose administration. However, the plasma insulin concentration data from saline-treated wild-type mice frequently failed to meet the quality control criteria (one of ten females; two of ten males; Table S1). Consequently, three-way ANOVA with genotype, sex and treatment (saline or glucose) as main effects could not be performed. To address this limitation, we used unpaired *t*-test to evaluate the sex difference of plasma insulin levels among heterozygous mice treated with saline but did not find significant differences (Fig. 3, left panel). However, two-way ANOVA was performed for mice treated with glucose to assess the effects of genotype, sex as well as any interaction between the two, revealed a significant genotype−sex interaction, as well as significant main effects of genotype and sex (Fig. 3, right panel). Post-hoc analysis using Šidák's multiple comparison test identified significant differences between (i) wild-type females vs heterozygous females, (ii) heterozygous females vs wild-type males and (iii) heterozygous females vs heterozygous males (Fig. 3, right panel). These results indicated a sex-dependent increase of plasma insulin levels following i.p. glucose administration.

**Fig. 3.**
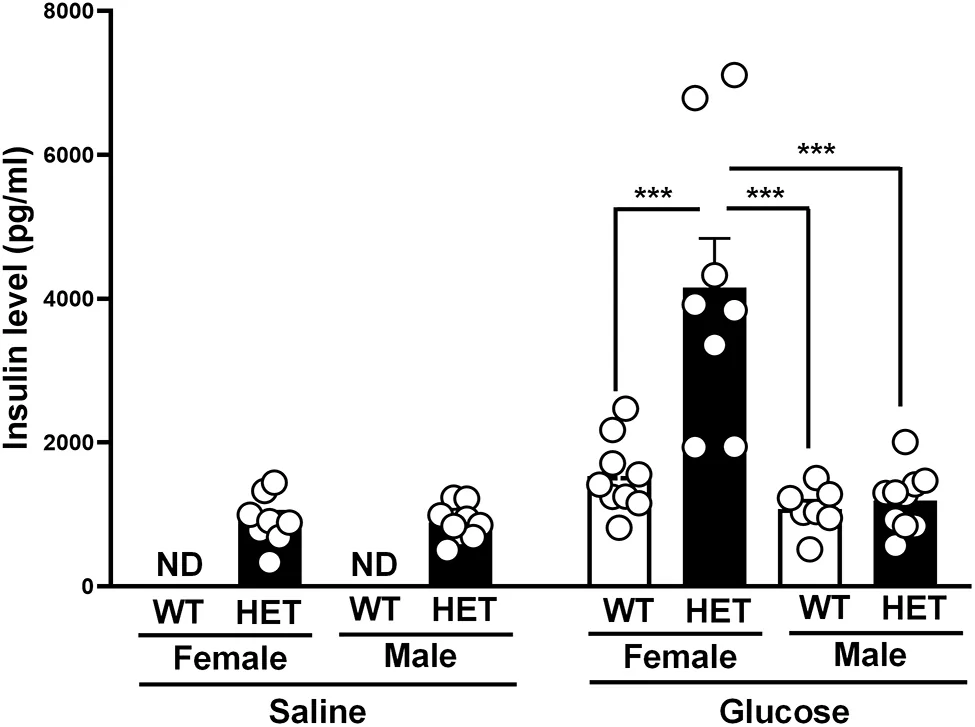
**Plasma insulin levels.** Bar graphs showing plasma insulin concentrations measured 15 min after intraperitoneal (i.p.) injection of saline (left) or glucose (right). Left: unpaired *t*-test for heterozygous (HET) female and male saline-treated mice [*t*=0.2151, d.f.=15 (with *t* denoting the *t*-statistic in Student's *t*-test and d.f. the degree of freedom), *P*=0.8326]. ND, no data available. Right: two-way ANOVA for glucose-treated mice. Interaction between genotype and sex (*F_1, 32_*=13.42, *P*=0.0009); effect of genotype (*F_1, 32_*=28.25, *P*<0.0001; with *F* denoting the *F*-statistic in ANOVA, and _*1*_ and _*31*_ the numerator and denominator degree of freedom, respectively); effect of sex (*F_1, 32_*=17.46, *P*=0.0002). Šidák's multiple comparisons test: wild-type (WT) female vs HET female mice (adjusted *P*<0.0001); WT female vs WT male mice (adjusted *P*=0.8239); WT female vs HET male mice (adjusted *P*=0.9535); HET female vs WT male mice (adjusted *P*<0.0001); HET female vs HET male mice (adjusted *P*<0.0001); WT male vs HET male mice (adjusted *P*=0.9995). Error bar represents the +standard error of the mean (+s.e.m.), individual values are indicated by circles. Sample sizes for saline-treated mice: HET females, *n*=8; HET males, *n*=9. Sample sizes for glucose-treated mice: WT females, *n*=9; WT males, *n*=7; HET females, *n*=8; HET males, *n*=10. ***, adjusted *P*<0.0001 in Šidák's multiple-comparisons test. The corresponding value for saline-treated WT mice is 0 because none of the mice in this group exceeded the cut-off value (see the Materials and Methods).

### Heterozygous mutants exhibited sexual dimorphism in body size and body fat ratio

In the dual-energy X-ray absorptiometry (DEXA) scan to measure bone density, fat mass and lean mass conducted at 22 weeks of age, body weight measurements revealed a significant interaction between genotype and sex (two-way ANOVA) (Fig. 4). Post-hoc analysis indicated a significant decrease in body weight observed only in heterozygous males (Šidák's multiple comparison test) (Fig. 4A). Body length measurements also showed significant effects of genotype and sex (two-way ANOVA), and post-hoc analysis indicated a significant decrease in body length only in heterozygous males (Šidák's multiple comparison test) (Fig. 4B). Additionally, in the same DEXA test, heterozygous females exhibited a significantly higher body fat ratio than wild-type females. Post-hoc analysis confirmed a significant difference in the body fat ratio between wild-type and heterozygous females (Šidák's multiple comparison test) (Fig. 4C).

**Fig. 4.**
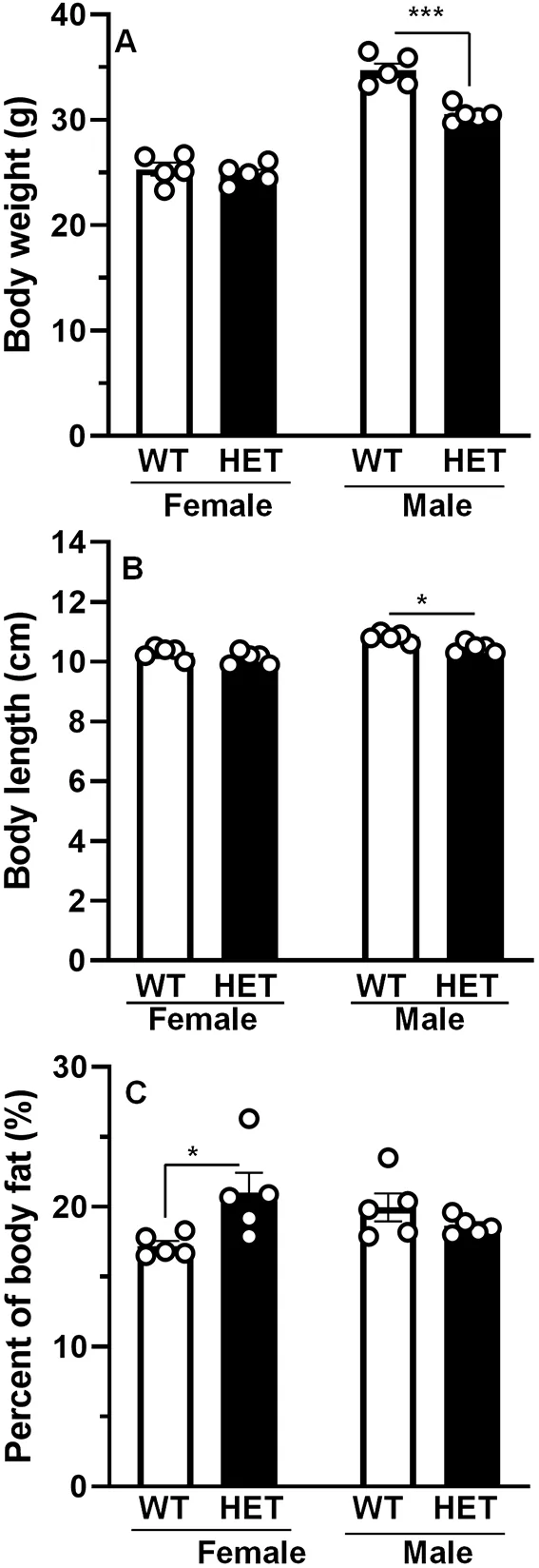
**DEXA scan results measuring fat mass and lean mass.** (A) Bar graphs represent the mean body weight in female and male wild-type (WT) and heterozygous (HET) mice. Two-way ANOVA: interaction between genotype and sex, *F_1, 16_*=12.51 (with *F* denoting the *F*-statistic in ANOVA, and _*1*_ and _*16*_ the numerator and denominator degree of freedom, respectively), *P*=0.0027; effect of genotype: *F_1, 16_*=19.50, *P*=0.0004; effect of sex: *F_1, 16_*=207.2, *P*<0.0001. Šidák's multiple-comparisons test: WT vs HET in females, adjusted *P*=0.79; WT vs HET in males, adjusted *P*<0.0001). (B) Bar graphs represent the mean body length in mice as indicated. Two-way ANOVA: interaction between genotype and sex, *F_1, 16_*=1.82, *P*=0.2930; effect of genotype: *F_1, 16_*=10.64, *P*=0.049; effect of sex: *F_1, 16_*=26.99, *P*<0.0001. Šidák's multiple-comparisons test: WT vs HET in females, adjusted *P*=0.2666; WT vs HET in males, adjusted *P*=0.0144. (C) Bar graphs represent the average body fat (in %) over whole-body mass in mice as indicated. Two-way ANOVA: interaction between genotype and sex, *F_1, 16_*=7.977, *P*=0.0122; effect of genotype: *F_1, 16_*=1.856, *P*=0.1920; effect of sex: *F_1, 16_*=0.04429, *P*=0.8360. Šidák's multiple-comparisons test, WT vs HET in females, adjusted *P*=0.0183; WT vs HET in males, adjusted *P*=0.5330. All error bars indicate the ±standard error of the mean (±s.e.m.); individual values are indicated by circles. Sample sizes were *n=*5 per group. *, adjusted *P*<0.05; ***, adjusted *P*<0.0001 in Šidák's multiple-comparisons test.

### Additional phenotypic features observed in heterozygous mutants in phenotyping pipeline 1

We conducted phenotyping pipeline 1 (PL1) testing on wild-type and heterozygous *Slc2a1^Rgsc200^* mice at the JMC. A summary of the statistical analyses of the data collected from PL1 is provided in Table S2. Additionally, as outlined under ‘Data and resource availability’, the data collected from PL1 of the mice in this study are accessible via the JMC website. In the phenotyping pipeline assessments, heterozygous mice exhibited no phenotypic changes compared with wild-type mice in the modified SHIRPA (a standardized assessment of neurological and behavioral functions), urinalysis (analysis of urinary biochemical and physical parameters) and funduscopy (examination of the ocular fundus) tests (Table S2). As described above, female *Slc2a1^Rgsc200^* mice exhibited significant differences regarding IPGTT and DEXA results. Additionally, Table S2 and Figs S1-S5 show experimental results for which statistically significant differences between genotypes were observed.

For hematological test data, i.e. white blood cell composition, including lymphocytes, monocytes and eosinophils, see Table S2, Fig. S1. For clinical blood chemistry data, i.e. increased levels of LDL-cholesterol, see Table S2, Fig. S2. For X-ray test data, i.e. tibia length reduction in limbs and morphological changes in vertebrae, see Table S2, Fig. S3. For electrocardiogram (ECG) data, i.e. coefficient of variation (CV), heart rate (HR), and RR interval, see Table S2, Fig. S4. For pathology data, i.e. right kidney weight, liver weight, body weight, see Table S2, Fig. S5. In addition, significant interactions between genotype and the relative weights of the spleen and whole body were observed. Post-hoc analysis revealed a significant effect of genotype on relative spleen weight in female mice. Finally, a significant interaction was observed between genotype, relative spleen weight and whole-body weight. Post-hoc analysis revealed a significant effect of genotype on the relative spleen weight, specifically in female mice (Fig. S5).

## DISCUSSION

### Significance of this study

In this study, we used the phenotyping PL1 at the JMC of RIKEN BRC to conduct comprehensive phenotyping of the *Slc2a1^Rgsc200^* Glut1DS mouse model. Heterozygous mutants of this strain did not exhibit changes in plasma glucose or HbA1c levels (Fig. 1A,B), which was consistent with previous findings in patients with Glut1DS (Klepper et al., 2020) and previously described animal models (Furuse et al., 2019; Wang et al., 2006). However, IPGTT revealed improved glucose tolerance in heterozygous females compared with wild-type females. The decrease of blood glucose levels began 30 min after glucose administration (Fig. 2A). Elevated insulin levels observed 15 min earlier were ∼2.6-fold higher in heterozygous females than in wild-type females, as measured independently by enzyme-linked immunosorbent assay (ELISA) (Fig. 3, right panel). As the mice used for insulin measurement were fasted for 16–18 h prior to blood collection (Fig. S6), insulin concentrations of saline-treated wild-type mice with outlier values were expected to fall below the detection limit (<136.53 pg/ml). However, 80–90% of the insulin concentration data from saline-treated heterozygote mice passed the quality control process. These findings suggest that baseline insulin levels in heterozygous mice were higher than those in wild-type mice. Moreover, basal insulin levels were comparable between heterozygous females and males (Fig. 3, left panel). Insulin, a crucial hormone secreted by the pancreas, facilitates cellular glucose uptake and decreases blood glucose levels (Saltiel and Kahn, 2001). The observed increase in circulating insulin levels is likely to account for the enhanced glucose tolerance observed in heterozygous females. In addition, we identified sex-specific metabolic phenotypes associated with elevated insulin levels. While a reduction in body size was observed exclusively in males (Fig. 4A,B), an increase in body fat levels was detected in females (Fig. 4C). The increase in body fat observed for heterozygous females may have resulted from altered insulin dynamics after food intake, which promotes lipid synthesis and facilitates glucose uptake. Although blood glucose levels remained unchanged in heterozygous mutants − similar to observation in Glut1DS patients and other mouse models, such as heterozygous *Slc2a1* knockout mice − alterations in glucose metabolism and insulin levels following food intake have so far not been thoroughly investigated in Glut1DS patients. Although sex differences regarding the incidence of Glut1DS have not been reported, our heterozygous mouse mutants exhibited notable sexual dimorphism across several phenotypes, including body size, body fat ratio, glucose metabolism and insulin levels, as described above. Previous studies have suggested that insulin levels in response to glucose administration are generally higher in females than in males (Horie et al., 2018; Kaikaew et al., 2019; Tramunt et al., 2020). Consequently, heterozygous females may exhibit heightened insulin responses to glucose stimulation during insulin level compared to heterozygous males, which could account for the observed sexual dimorphism. A previous study found that brain astrocyte-specific deletion of the *Slc2a1* gene in mice that had been fed a high-fat diet results in reduced blood glucose levels and increased circulating insulin levels following glucose administration (Ardanaz et al., 2024). Additionally, this conditional mutant shows elevated insulin receptor expression in astrocytes (Ardanaz et al., 2024). Given that Ardanaz et al. had induced deletion of *Slc2a1* in astrocytes by tamoxifen administration to mice aged 8 weeks and focused on the role of SLC2A1 and insulin signaling in brain energy metabolism, our *Slc2a1^Rgsc200^* mouse strain shares only limited commonality with this astrocyte-specific model. However, the findings our current study suggest that altered insulin signaling can also occur in *Slc2a1^Rgsc200^* mutants. This suggests that alterations in insulin pathways may be a broader feature of GLUT1 deficiency syndromes, warranting further investigation. Recent evidence indicates that managing elevated blood glucose levels through intake of the insulin inhibitor diazoxide, together with continuous glucose monitoring, may improve clinical outcomes for Glut1DS patients who are resistant to therapy with KD (Logel et al., 2021). These findings underscore the potential role of individual differences in glucose metabolism and insulin secretion in the therapeutic resistance to the KD. Further studies are needed to investigate glucose and insulin dynamics in Glut1DS patients to elucidate the mechanisms of KD resistance, and to identify effective therapeutic interventions. The novel findings of this study provide crucial insights into the Glut1DS mouse model, particularly the role of altered insulin dynamics in glucose metabolism and their potential sex-specific effects. These new findings provide important insights to the Glut1DS mouse model, particularly regarding the role of altered insulin dynamics during glucose metabolism and the potential sex-specific effects of these dynamics. Our results contribute substantially to the understanding of metabolic phenotypes of Glut1DS and underscore the therapeutic potential of insulin-inhibiting pharmaceuticals, as demonstrated in clinical case studies (Logel et al., 2021). Additionally, because peripheral plasma insulin levels are influenced not only by pancreatic insulin secretion but also by insulin clearance − particularly hepatic insulin extraction − further studies to measure circulating C-peptide levels or glucose-stimulated insulin secretion in isolated islets are required to determine whether these changes directly reflect altered β-cell insulin secretory function.

In our current study, unbiased, comprehensive screening by using a mouse phenotyping PL1 identified several phenotypic changes in the *Slc2a1^Rgsc200^* mutant mouse, in addition to alterations in glucose metabolism and insulin secretion. They included changes in white blood cell composition, elevated low-density lipoprotein (LDL) cholesterol levels, skeletal morphological abnormalities, altered ECG wave patterns and differences in organ weight (Table S2; Figs S1-5). The coefficient of variation (CV) (Fig. S4C) was calculated from the RR intervals as the standard deviation divided by the mean RR interval and used as an indicator of beat-to-beat variability in cardiac rhythm. Higher CV values indicated greater variability in the RR intervals, whereas lower CV values indicated reduced beat-to-beat variability. Although subtle, these findings warrant further investigation to enhance our understanding of the pathogenesis of Glut1DS.

### Limitations of this study

Although the effectiveness of the insulin inhibitor diazoxide in managing Glut1DS symptoms had been demonstrated previously (Logel et al., 2021) and our current study supports the importance of these findings, we did not directly test diazoxide in this study. Furthermore, mutations in *SLC2A1*, which causes Glut1DS symptoms, exhibit substantial variation. To address these limitations, it is essential to expand the repertoire of animal models and conduct preclinical studies to evaluate the role of insulin signaling as a potential therapeutic target in Glut1DS. Clinical manifestations of Glut1DS change with age. In patients, epileptic symptoms are typically more prominent during infancy and childhood; seizure frequency often decreases with age, and movement-related abnormalities, including paroxysmal exercise-induced dyskinesia and motor dysfunction, may become more apparent during adolescence and adulthood (Leen et al., 2014; Olivotto et al., 2022). In our current study, mice aged 8–26 weeks were longitudinally analyzed. These testing ages approximately correspond to the stages of young adults to mature adults in mice. Therefore, our measurements are likely to reflect relatively stable adult-phase phenotypes rather than early developmental manifestations analogous to infantile epileptic encephalopathy in humans.

### Conclusion

This study underscores the utility of comprehensive phenotyping platforms to uncover subtle, yet significant, phenotypes in the Glut1DS model. By integrating metabolic and systemic assessments, our findings provide a foundation for the development of targeted therapies to address the diverse manifestations of Glut1DS.

## MATERIALS AND METHODS

### Animal experiments − ethics statement

All procedures described in this study have been reviewed and approved by the Institutional Animal Care and Use Committee of RIKEN Tsukuba Branch (approval no.: T2024-EP006).

### Glut1DS mouse model

The experimental conditions for mouse husbandry were carefully controlled as follows: air temperature was maintained between 20 and 24°C, humidity between 45 and 60% and lighting was regulated under a 12:12 h light:dark cycle, with lights on at 8:00 a.m. All mice had *ad libitum* access to food (CE2; CLEA Japan, Inc., Tokyo, Japan) and distilled water. As previously reported (Furuse et al., 2019), the B6(D2)-Slc2a1<Rgsc200>/1Rbrc mutant strain is established as a model for Glut1DS. This strain was developed using a large-scale mutagenesis program conducted at the RIKEN Genomic Sciences Center at the ENU. Genetic analysis revealed that the strain carries a thymine-to-cytosine point mutation within exon 7 of *Slc2a1*, resulting in a Ser324Pro substitution in SLC2A1. Initially designated *Glut1^Rgsc200^*, the mutation is now officially referred to as *Slc2a1^Rgsc200^*. Mice for this study were produced by *in vitro* fertilization and embryo transfer using sperm from heterozygous males and oocytes from C57BL/6JJcl females purchased from a commercial supplier (CE2, CLEA Japan, Inc.), as previously described by Furuse et al. (2017). The nutrient composition of the CE-2 diet is available on the supplier's website (https://www.clea-japan.com/en/products/general_diet/item_d0030). Given that homozygous mutants exhibit embryonic lethality (Furuse et al., 2019), we used wild-type and heterozygous mice in our current study. Mice used for plasma insulin measurements were generated independently from the mice used in the phenotyping pipeline.

### Comprehensive phenotyping of the Glut1DS mouse model

The *Slc2a1^Rgsc200^* mutant mice were phenotypically characterized by using the comprehensive phenotyping pipeline (PL1) at the JMC of the RIKEN BRC. The phenotyping PL1 and detailed experimental procedures are described on the JMC website (https://jmc-en.brc.riken.jp/mouse_clinic/SOPs/index_mc.html); methodology has been described previously (Hirata et al., 2022; Hirose et al., 2017). The age of mice when conducting each test is listed in Table 1. Whole blood samples for clinical blood chemistry and HbA1c measurements were collected from mice at fed state, i.e. without a defined fasting period.

**Table 1.** Overview of performed tests, listing age and number of mice per experimental group subjected to a comprehensive phenotyping pipeline at the Japan Mouse Clinic

Test	Age [weeks]	Number			
		Female		Male	
		Wild type	Heterozygote	Wild type	Heterozygote
Modified SHIRPA	8	7	7	7	7
Hematology	9	7	7	7	7
Urinalysis	10	7	7	7	7
Clinical blood chemistry	11	7	7	7	7
IPGTT	14	7	7	7	7
Funduscopy	19	5	5	5	5
X-ray	22	7	7	7	7
DEXA scan	22	5	5	5	5
ECG	24	5	5	5	5
Pathology	26	3	5	3	5

Heterozygote, heterozygous mutant of the B6(D2)-Slc2a1<Rgsc200>/1Rbrc; Modified SHIRPA, a standardized assessment of neurological and behavioral functions; Hematology, analysis of blood cell counts and related hematological parameters; Urinalysis, analysis of urinary biochemical and physical parameters; Clinical blood chemistry, analysis of biochemical parameters in blood, IPGTT, intraperitoneal glucose tolerance test; Funduscopy, examination of the ocular fundus, including the retina and optic disc; X-ray, radiographic examination of skeletal structures; DEXA scan, dual-energy X-ray absorptiometry for measurement of bone density, fat mass and lean mass; ECG, electrocardiography for assessment of the electrical activity of the heart, including heart rate and rhythm; Pathology, gross and histopathological examination of tissues and organs.

### Quality control procedure of clinical blood chemistry tests

Plasma samples with a hemolysis index exceeding the quality control threshold (>0.5), as measured using a clinical blood chemistry test kit (KANTO CHEMICAL Co., Inc., Tokyo, Japan), were excluded from the analyses of hemolysis-sensitive parameters.

### Intraperitoneal glucose tolerance test

As the primary focus of this study was on glucose metabolism in the Glut1DS mouse model, the detailed procedure of the intraperitoneal glucose tolerance test (IPGTT) conducted on PL 1 at the JMC was as follows: ∼16–18 h before the test, the cages, water bottles, and bedding were replaced. During this period, the animals were housed in food-deprived cages to maintain an overnight fasting state while water remained available. Following the fasting period, mice were weighed and intraperitoneally injected with 20% glucose solution in 0.9% NaCl (glucose dose: 2 g/kg body weight). Blood samples were collected from tail tips. A minimal blood volume (<5 μl) was applied to a test strip (G-sensor, ARKRAY, Inc., Kyoto, Japan) and measured by using a glucose meter (GLUCOCARD G+ Meter GT-1820, ARKRAY, Inc.) to determine glucose levels. After measuring the baseline blood glucose level (denoted as 0 min in Fig. 2A,B and Fig. S7), glucose solution was administered, and blood glucose levels were measured for each mouse 15, 30, 60 and 120 min post injection. A detailed experimental timeline is provided in Fig. S7; age and number of subjects used are summarized in Table 1.

### Measurement of plasma insulin levels after intraperitoneal glucose administration

Fourteen- to fifteen-week-old mice were fasted for ∼16–18 h, and glucose or saline solution was administered intraperitoneally as described in the IPGTT section above. Fifteen minutes after administration, mice were deeply anesthetized with 3% isoflurane gas. Blood samples (∼200 μl) were collected from the retro-orbital sinus with a Pasteur pipette (Thermo Fisher Scientific, Waltham, MA, USA). The blood collection time was determined based on the study by Marguet et al. (2000). The collected blood was transferred to heparin-lithium-coated microtubes (BD Microtainer; Becton, Dickinson and Company, Franklin Lakes, NJ, USA) and placed on ice. Blood samples were centrifuged twice (at 5000***g*** at 8°C) for 10 min and 5 min to separate the plasma, which was stored at −80°C for subsequent analysis. A detailed experimental timeline is provided in Fig. S6.

### Insulin measurement

Plasma insulin levels were measured by enzyme-linked immunosorbent assay (ELISA) with the Bio-Plex Pro Mouse Diabetes Insulin Set (#171G7006M; Bio-Rad Laboratories, Inc., Hercules, CA, USA). Measurements were performed using the Bio-Plex Pro Reagent Kit V (#12002798, Bio-Rad Laboratories, Inc.) and the Bio-Plex 200 system (Bio-Rad Laboratories, Inc.) following the manufacturer's protocol. Each sample was analyzed in duplicate. The quality control process for insulin measurement was conducted as follows: duplicate data in which both values fell within the measurement range of 136.53–110, 407.34 pg/ml, as calculated by the Bio-Plex Manager Software (Bio-Rad Laboratories, Inc.), were averaged and the mean values were used for statistical analyses.

### Statistical analysis

For the statistical analyses of the phenotyping results, we employed unpaired *t*-test (factor: sex) and two-way ANOVA (factors: genotype and sex). Šidák's multiple comparison test was conducted as a post-hoc analysis following a two-way ANOVA. Differences were considered statistically significant at *P*<0.05 for ANOVA results, and at adjusted *P*-values for post-hoc tests. All statistical analyses were conducted using the GraphPad Prism 8 software (GraphPad Software, LLC, MA, USA). To compare assay results expressed in nominal scales, Pearson's chi-squared (χ^2^) test was conducted by using Mou-Stat software (Suzuki et al., 2013).

## Supplementary Material

10.1242/dmm.052836_sup1 Supplementary information
