# Supplementary material for: Female-specific alterations in insulin dynamics are associated with glucose tolerance in a Glut1DS mouse model
Source: Dis Model Mech. 2026 Sep 21;19(9):dmm052836. doi: 10.1242/dmm.052836 (PMC13651954; doi:10.1242/dmm.052836)
Supplement: Supplementary file 1 [file dmm-19-052836-s1.pdf]

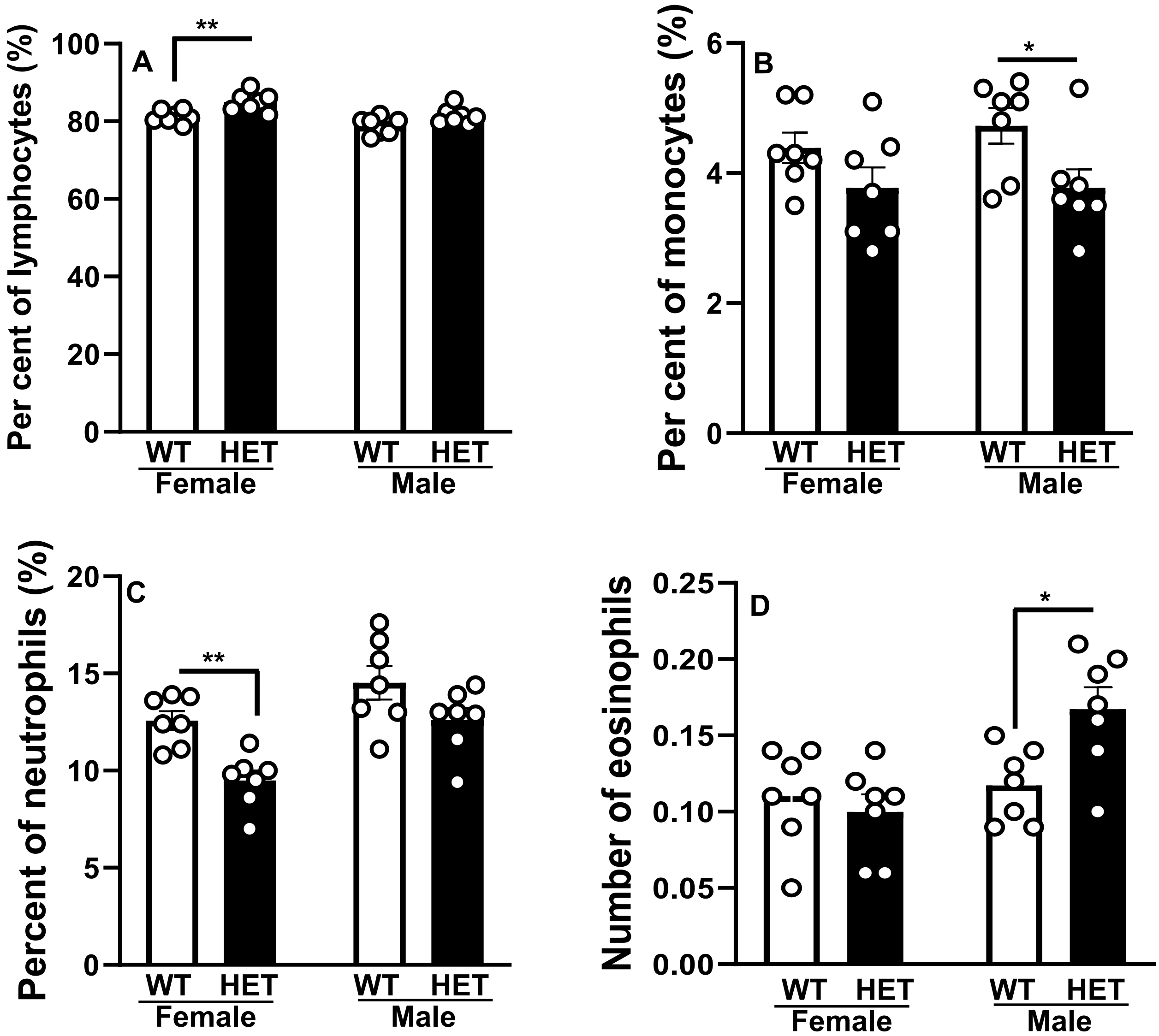

Fig. S1. Hematological test.

(A) Percent of lymphocytes. The mean lymphocyte percentage for each group is shown. Two-way ANOVA: interaction between genotype and sex, $F_{1, 24}$ = 0.6103, P = 0.4423; effect of genotype, $F_{1, 24}$ = 15.82, P = 0.0006; effect of sex, $F_{1, 24}$ = 13.49, P = 0.0012. Sidak's multiple-comparisons test: wildtype vs. heterozygote in females, *Adjusted P* = 0.0051; wildtype vs. heterozygote in males, *Adjusted P* = 0.0652.

(B) Per cent of monocytes. The mean monocyte percentage for each group is shown. Two-way ANOVA: interaction between genotype and sex, $F_{1, 24}$ = 0.3748, P = 0.5461; effect of genotype, $F_{1, 24}$ = 7.874, P = 0.0098; effect of sex, $F_{1, 24}$ = 0.3748, P = 0.5461. Sidak's multiple-comparisons test: wildtype vs. heterozygote in females, *Adjusted P* = 0.5461; wildtype vs. heterozygote in males, *Adjusted P* = 0.0467.

(C) Per cent of neutrophils. The mean neutrophil percentage for each group is shown. Two-way ANOVA: interaction between genotype and sex, $F_{1, 24}$ = 0.8146, P = 0.3757; effect of genotype, $F_{1, 24}$ = 15.3, P = 0.007; effect of sex, $F_{1, 24}$ = 16.65, P = 0.0006. Sidak's multiple-comparisons test: wildtype vs. heterozygote in females, *Adjusted P* = 0.0047; wildtype vs. heterozygote in males, *Adjusted P* = 0.0858.

(D) Absolute number of eosinophils. The mean eosinophil count for each group is shown. Two-way ANOVA: interaction between genotype and sex, $F_{1, 24}$ = 6.330, P = 0.0190; effect of genotype, $F_{1, 24}$ = 2.813, P = 0.1065; effect of sex, $F_{1, 24}$ = 9.703, P = 0.0047. Sidak's multiple-comparisons test: wildtype vs. heterozygote in females, *Adjusted P* = 0.8053; wildtype vs. heterozygote in males, *Adjusted P* = 0.0134.

(A) – (D), error bars represent the standard error of the mean (SEM), and individual values are indicated by circles. Sample sizes: N = 7 per group. WT, wildtypes; HET, heterozygotes. *, *Adjusted P* < 0.05; **, *Adjusted P* < 0.01 in Sidak's multiple-comparisons test.

Disease Models & Mechanisms • Supplementary information

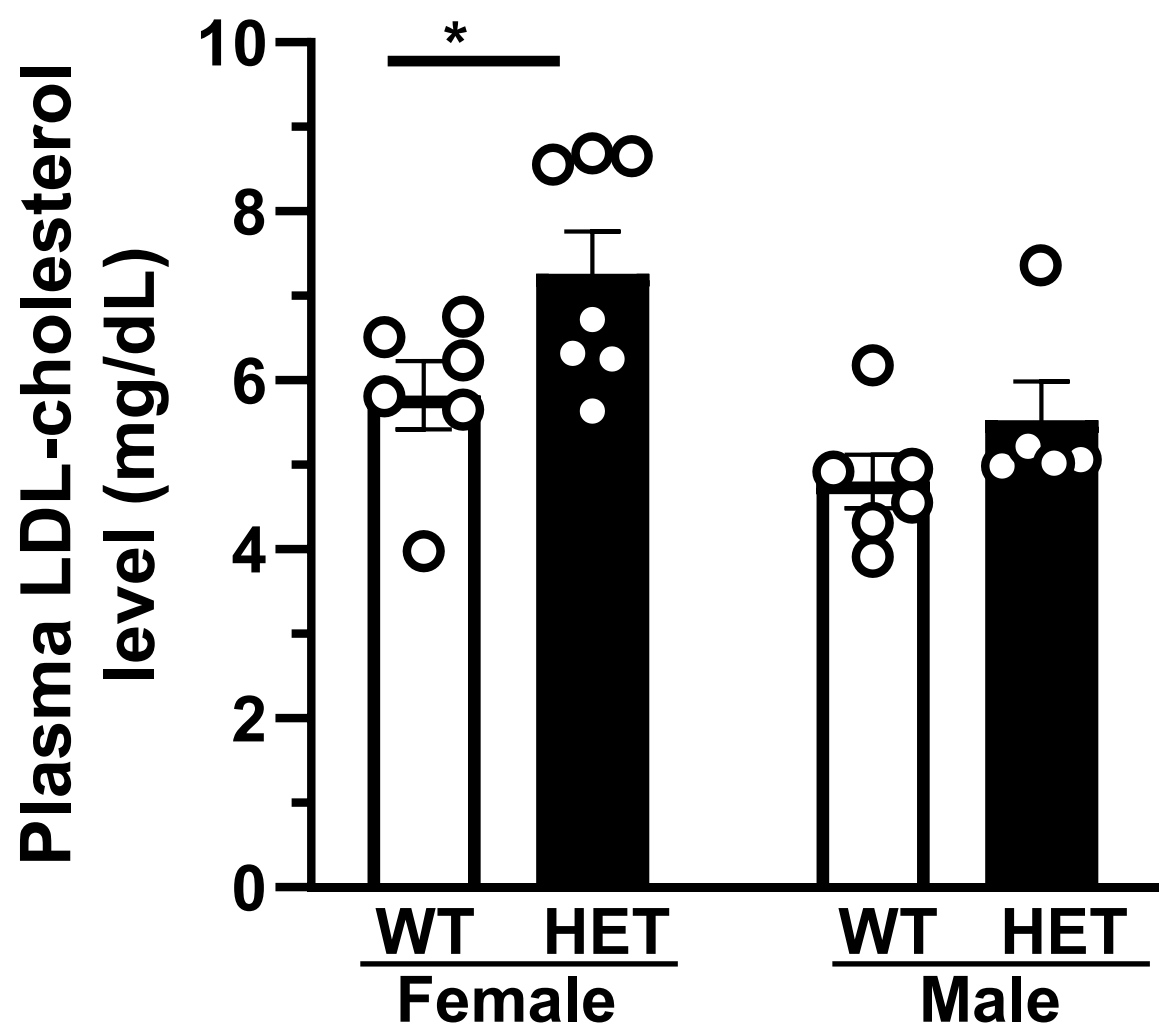

Fig. S2. Low density lipoprotein (LDL) cholesterol in clinical blood chemistry test.

The mean LDL cholesterol level in plasma for each group is shown. Two-way ANOVA: interaction between genotype and sex, $F_{1, 20}$ = 0.6584, P = 0.4267; effect of genotype, $F_{1, 20}$ = 6.112, P = 0.0225; effect of sex, $F_{1, 20}$ = 9.877, P = 0.0051. Sidak's multiple-comparisons test: wildtype vs. heterozygote in females, *Adjusted P* = 0.490; wildtype vs. heterozygote in males, *Adjusted P* = 0.4711. *, *Adjusted P* < 0.05 in Sidak's multiple-comparisons test. Error bars represent the standard error of the mean (SEM), and individual values are indicated by circles. Sample sizes for saline-treated mice: heterozygous females, N = 8; heterozygous males, N = 9. Sample sizes: wild-type females, N = 6; wild-type males, N = 6; heterozygous females, N = 8; heterozygous males, N = 6. WT, wildtypes; HET, heterozygotes.

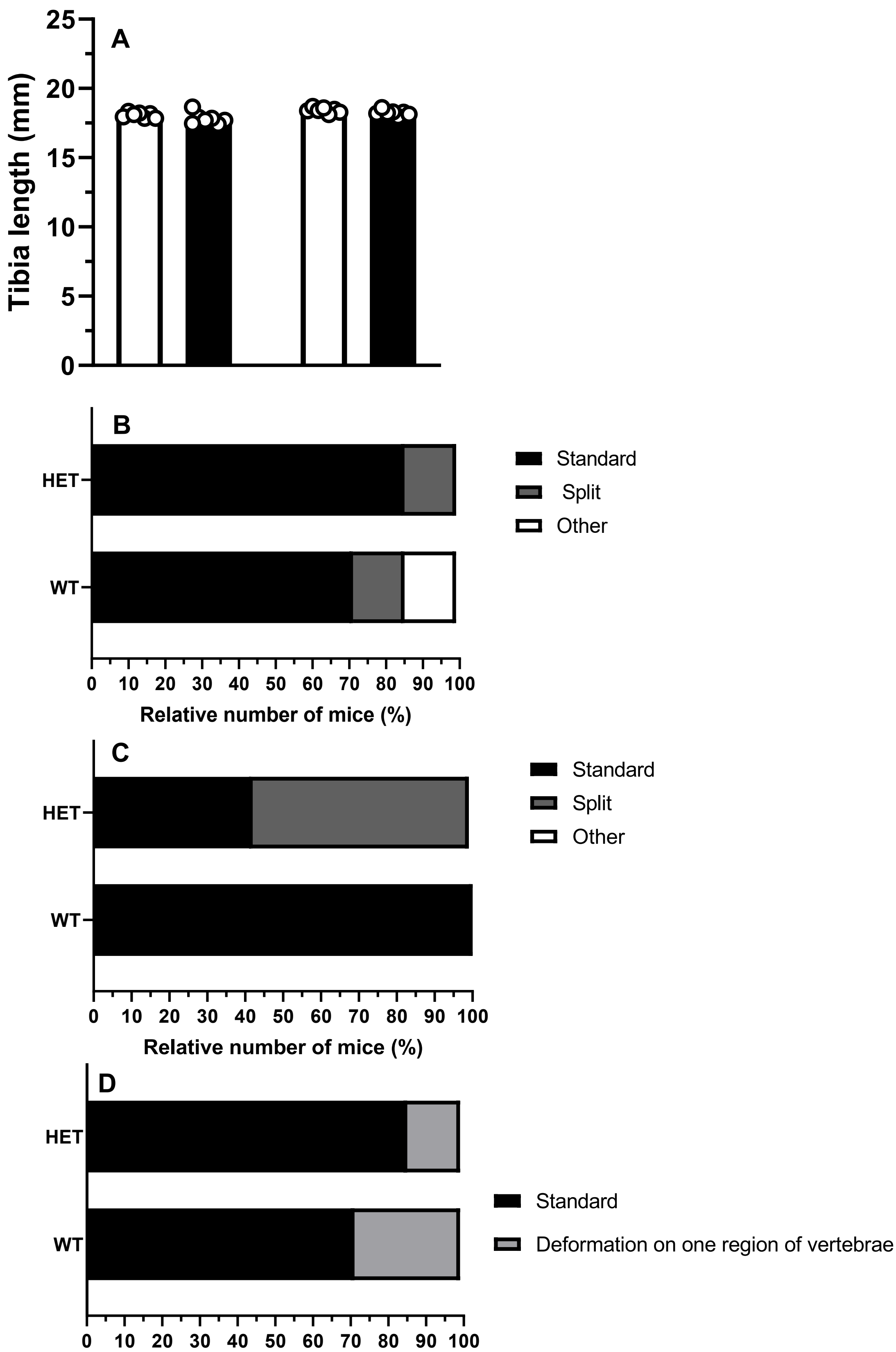

A
Tibia length (mm)
0
5
10
15
20
25
B
HET
WT
Standard
Split
Other
0 10 20 30 40 50 60 70 80 90 100
Relative number of mice (%)
C
HET
WT
Standard
Split
Other
0 10 20 30 40 50 60 70 80 90 100
Relative number of mice (%)
D
HET
WT
Standard
Deformation on one region of vertebrae
0 10 20 30 40 50 60 70 80 90 100

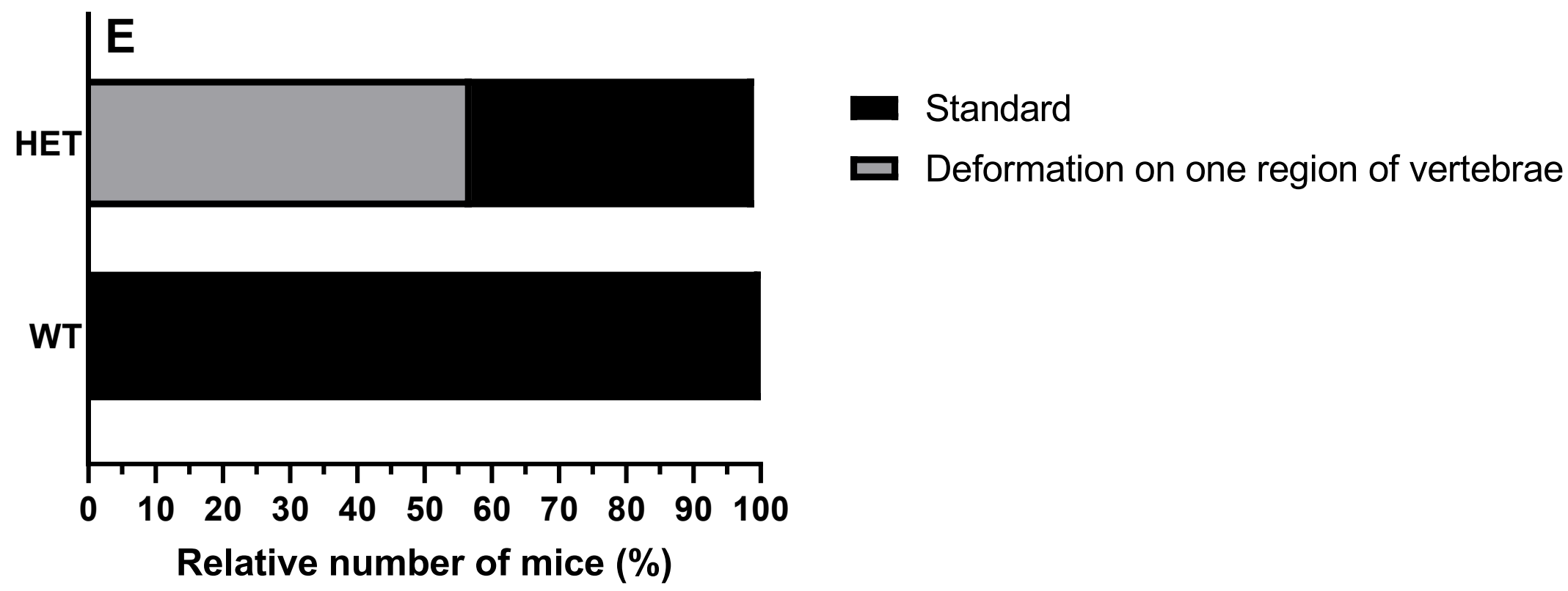

Fig. S3. X-ray test.

Results of X-ray imaging test that demonstrated statistical significance are shown.

(A) Tibia length. The mean tibia length for each group is shown. Two-way ANOVA: interaction between genotype and sex, $F_{1, 24}$ = 0.1988, *P* = 0.6597; effect of genotype, $F_{1, 24}$ = 4.462, *P* = 0.0453; effect of sex, $F_{1, 24}$ = 15.14, *P*=0.0007. Error bars represent the standard error of the mean (SEM), and individual values are indicated by circles.

(B) The percentage distribution of mice based on cervical vertebrae shape is shown. *Pearson's chi-square test*: χ^2 = 1.091, *df* = 2, *P* = 0.5796.

(C) Shape of cervical vertebrae in males. The percentage distribution of mice based on cervical vertebrae shape is shown. *Pearson's chi-square* test: χ^2 = 5.600, *df* = 1, *P* = 0.01796.

(E) Shape of vertebrae in females. The percentage distribution of mice based on vertebrae shape is shown. *Pearson's chi-square* test: χ^2 = 1.091, *df* = 2, *P* = 0.5796.

(F) Shape of vertebrae in males. The percentage distribution of mice based on vertebrae shape is shown. *Pearson's chi-square* test: χ^2 = 5.600, *df* = 1, *P* = 0.01796.

(A) - (F) Sample sizes: *N* = 7 per group. WT, wildtypes; HET, heterozygotes.

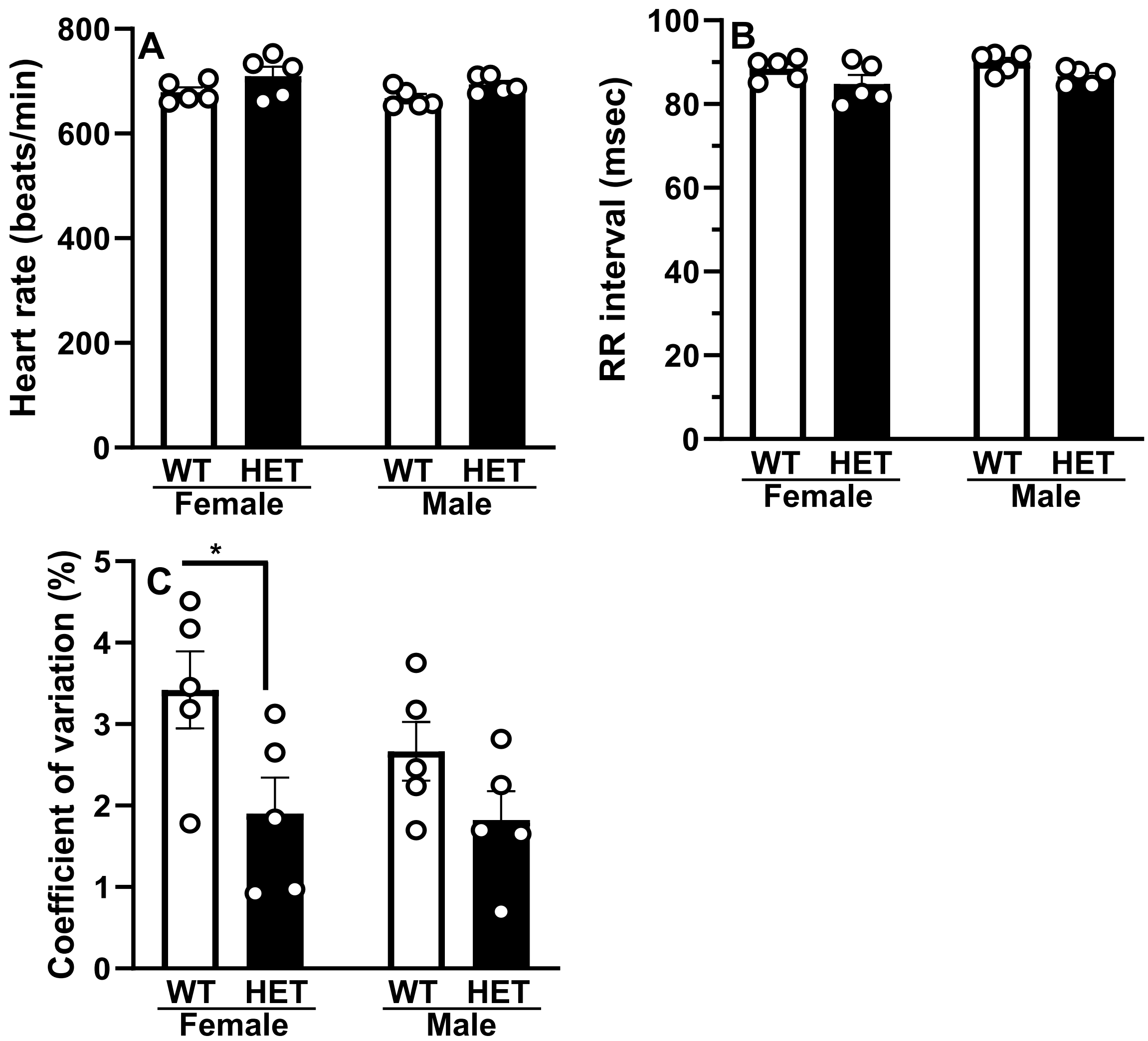

Fig. S4. Electrocardiogram (ECG) test.

(A) Heart rate. The mean heart rate for each group is presented. Two-way ANOVA: interaction between genotype and sex, $F_{1, 16}$ = 0.6798, *P* = 0.4218; effect of genotype, $F_{1, 16}$ = 8.275, *P* = 0.0110; effect of sex, $F_{1, 16}$ = 1.032, *P*=0.3249. Sidak's multiple-comparisons test: wildtype vs. heterozygote in females, *Adjusted P* = 0.0370; wildtype vs. heterozygote in males, *Adjusted P* = 0.3046. *, *Adjusted P* < 0.05 in Sidak's multiple-comparisons test.

(B) RR interval. The mean RR interval (interval between the peaks of consecutive R waves) for each group is presented. Two-way ANOVA: interaction between genotype and sex, $F_{1, 16}$ = 0. 04032, *P* = 0.8434; effect of genotype, $F_{1, 16}$ = 6.176, *P* = 0.0244; effect of sex, $F_{1, 16}$ = 1.507, *P*=0.2373. *Sidak's* multiple-comparisons test: wildtype vs. heterozygote in females, *Adjusted P* = 0.1457; wildtype vs. heterozygote in males, *Adjusted P* = 0. 2357.

(C) Coefficient of variation (CV). The mean CV, calculated from the standard deviation (SD) of the RR intervals for each mouse as a percentage, is presented. Two-way ANOVA: interaction between genotype and sex, $F_{1, 16}$ = 0. 007809, *P* = 0.9307; effect of genotype, $F_{1, 16}$ = 6.175, *P* = 0.0244; effect of sex, $F_{1, 16}$ = 1.406, *P*=0.2531. Sidak's multiple-comparisons test: wildtype vs. heterozygote in females, *Adjusted P* = 0.1675; wildtype vs. heterozygote in males, *Adjusted P* = 0.2070.

(A) - (C) Error bars represent the standard error of the mean (SEM), and individual values are indicated by circles. Sample sizes: *N*=5 per group.WT, wildtypes; HET, heterozygotes.

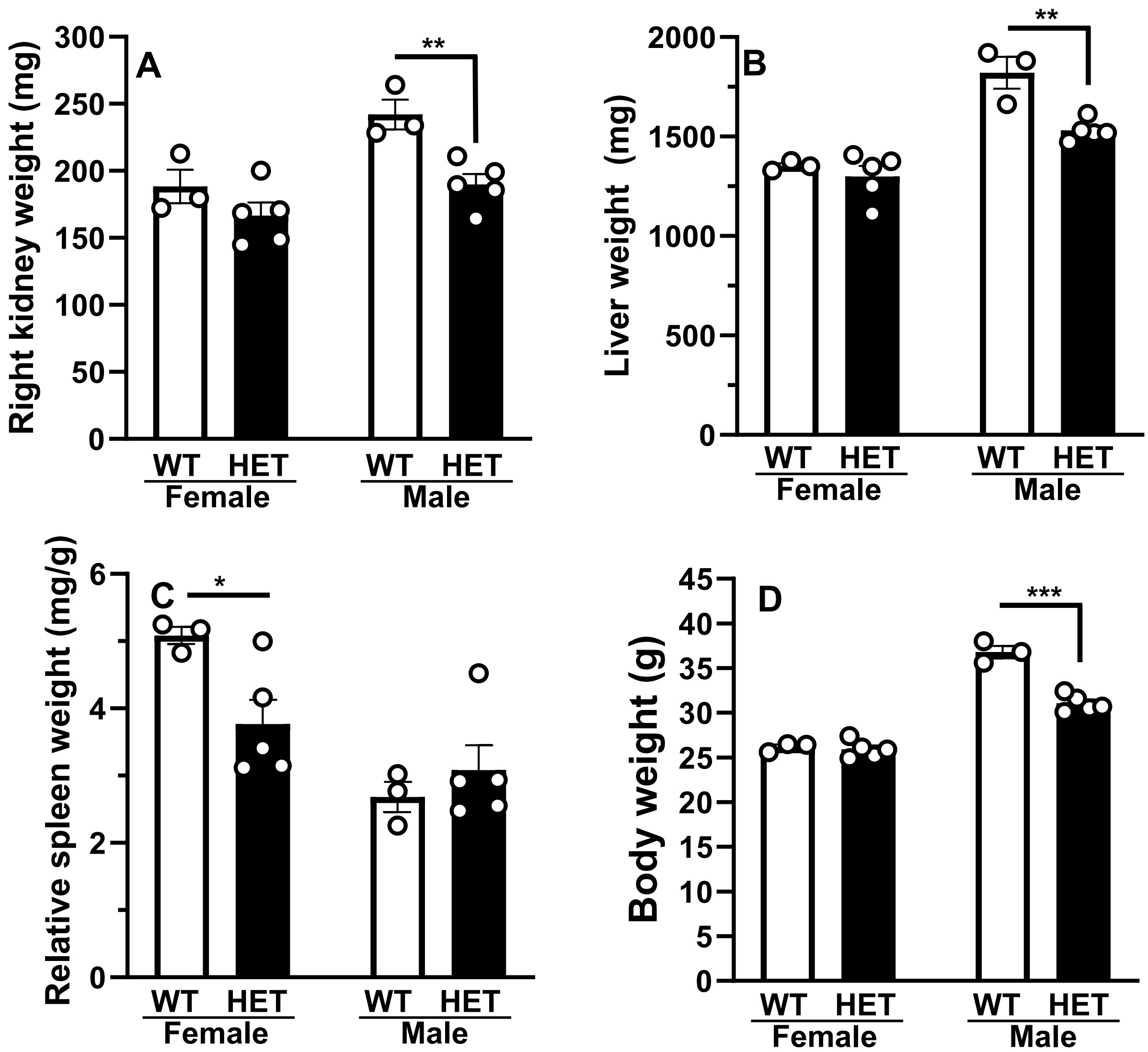

Fig. S5. Gross pathology test.

(A) Right kidney weight. Mean wight of right kidney for each group is presented. Two-way ANOVA: interaction between genotype and sex, $F_{1, 12}$ = 2.181, P = 0.1655; effect of genotype, $F_{1, 12}$ = 12.81, P = 0.0038; effect of sex, $F_{1, 12}$ = 13.90, P = 0.0029. Sidak's multiple-comparisons test: wildtype vs. heterozygote in females, *Adjusted P* = 0.2991; wildtype vs. heterozygote in males, *Adjusted P* = 0.0076.

(B) Liver weight. Mean liver wight for each group is presented. Two-way ANOVA: interaction between genotype and sex, $F_{1, 12}$ = 5.881, P = 0.0320; effect of genotype, $F_{1, 12}$ = 12.27, P = 0.0320; effect of sex, $F_{1, 12}$ = 51.27, P < 0.0001. Sidak's multiple-comparisons test: wildtype vs. heterozygote in females, *Adjusted P* = 0.7090; wildtype vs. heterozygote in males, *Adjusted P* = 0.0025.

(C) Relative spleen weight. Mean spleen weight relative to whole body weight for each group is presented. Two-way ANOVA: interaction between genotype and sex, $F_{1, 12}$ = 5.755, P = 0.0336; effect of genotype, $F_{1, 12}$ = 1.652, P = 0.229; effect of sex, $F_{1, 12}$ = 18.58, P < 0.0010. Sidak's multiple-comparisons test: wildtype vs. heterozygote in females, *Adjusted P* = 0.455; wildtype vs. heterozygote in males, *Adjusted P* = 0.6934.

(D) Body weight. Mean body wight for each group is presented. Two-way ANOVA: interaction between genotype and sex, $F_{1, 12}$ = 31.8 P = 0.0001; effect of genotype, $F_{1, 12}$ = 38.3, P < 0.0001; effect of sex, $F_{1, 12}$ = 264.8, P < 0.0001. Sidak's multiple-comparisons test: wildtype vs. heterozygote in females, *Adjusted P* = 0.9126; wildtype vs. heterozygote in males, *Adjusted P* < 0.0001.

(A) – (D), error bars represent the standard error of the mean (SEM), and individual values are indicated by circles. Sample sizes: wildtype, N = 3 per sex; heterozygote, N = 5 per sex. WT, wildtypes; HET, heterozygotes. *, Adjusted P < 0.05; **, Adjusted P < 0.01; ***, Adjusted P < 0.0001 in *Sidak's multiple-comparisons* test.

Disease Models & Mechanisms • Supplementary information

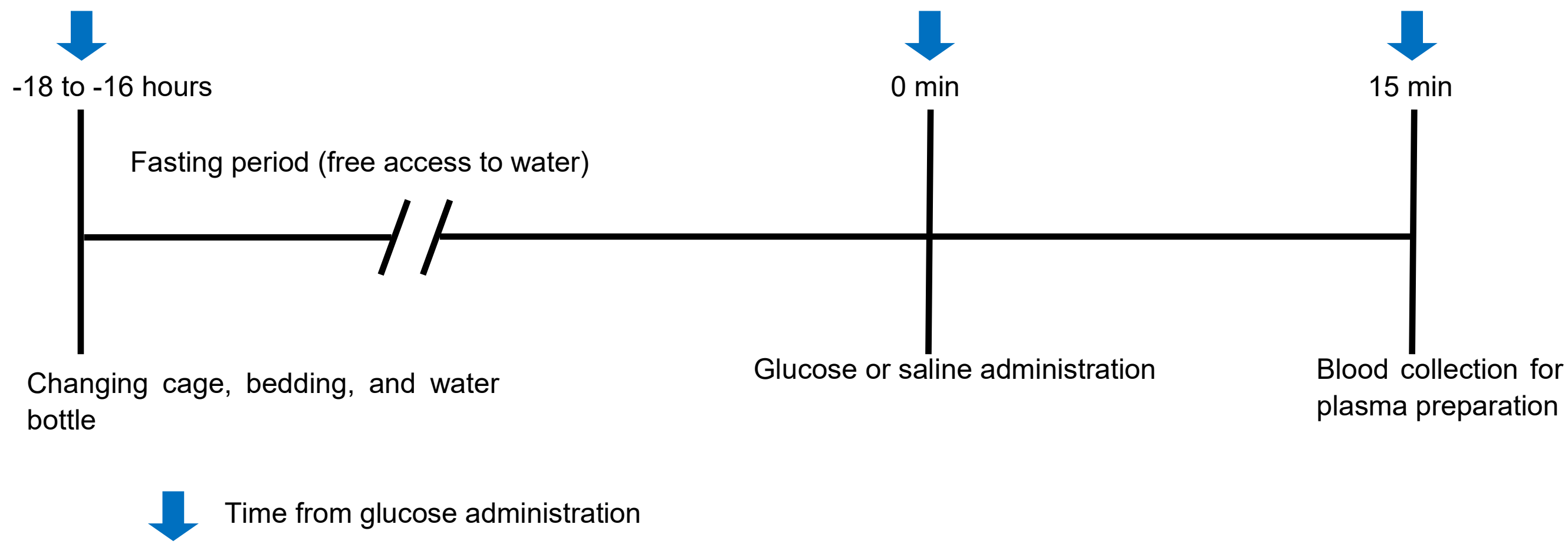

Fig. S6. Timeline of blood collection for insulin measurement.

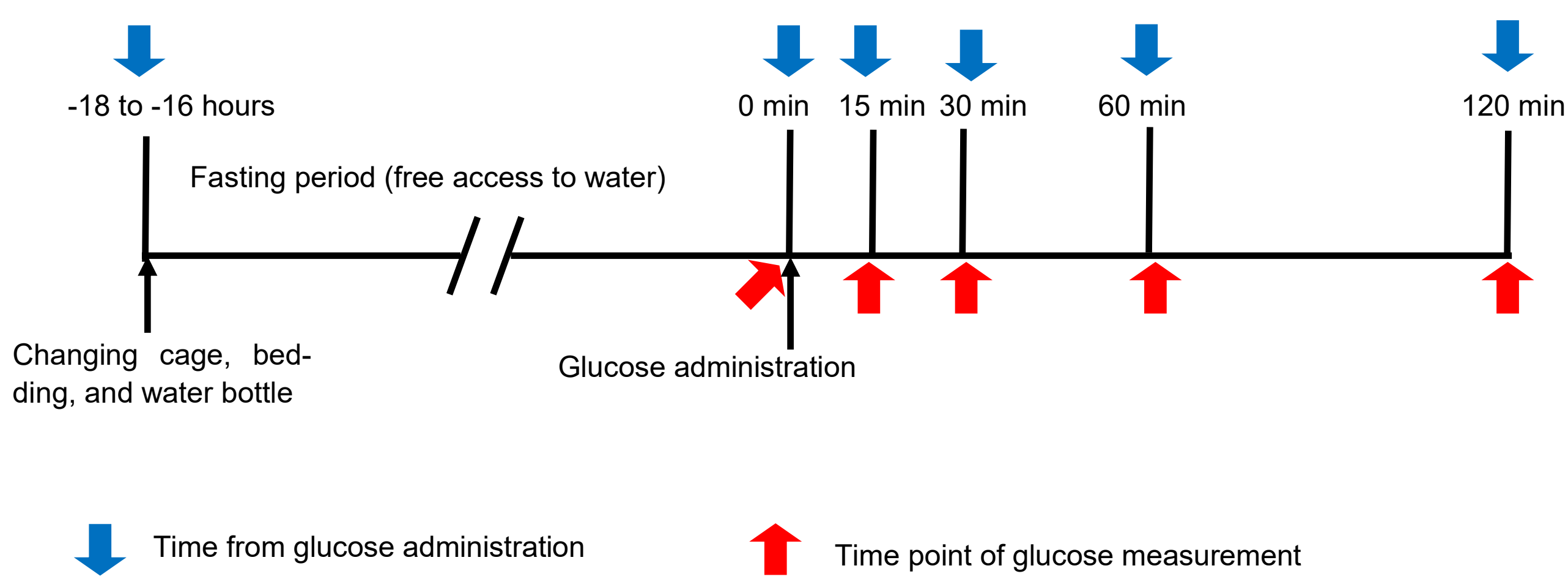

Fig. S7. Timeline of intraperitoneal glucose tolerance test (IPGTT).

Disease Models & Mechanisms • Supplementary information

Table S1 . Results of Data Quality Control for Plasma Insulin Measurement.

	WT								HET							
	Female				Male				Female				Male			
	PQC	NPC	Total	% of NPC	PQC	NPC	Total	% of NPC	PQC	NPC	Total	% of NPC	PQC	NPC	Total	% of NPC
Saline	1	9	10	90.0%	2	8	10	80.0%	8	2	10	20.0%	9	1	10	10.0%
Glucose	10	0	10	0.0%	8	2	10	20.0%	8	2	10	20.0%	10	0	10	0.0%

The numbers or percentages of data that passed or failed the quality control process described in the materials and methods section are represented.

WT, wild type; HET, heterozygote.

PQC, Data that Passed Quality Control Criteria; NPC, Data that did Not Pass Quality Control Criteria.

Disease Models & Mechanisms • Supplementary information

Table S2. Statistical summary of the data collected in the PL1 in the Japan mouse clinic

Age (weeks)	Procedure	Parameter	Sub-parameter	Statistical significance
8	Modified-SHIRPA	Abdominal tone	-	NS
		Activity in novel environment	Time elapsed before the mouse starts to move	NS
			Locomotor activity	NS
			Transfer arousal	NS
		Aggression	-	NS
		Body size	Body mass index (BMI)	NS
			Body weight	NS
			Body length	NS
			Tail length	NS
			Tail ratio	NS
		Coat color	Major color	NS
			Minor color	NS
			Pattern in the region with a different color	NS
			White belly	NS
		Contact righting	-	NS
		Corneal reflex	-	NS
		Eye	Cornea	NS
			Eye color	NS
			Eye shape	NS
			Eye size	NS
			Pupil	NS
		Fear behavior	-	NS
		Gait	-	NS
		Hair length	-	NS
		Hair morphology	-	NS
		Head morphology	-	NS
		Heart rate	-	NS
		Irritability behavior	-	NS
		Lacrimation	-	NS
		Limb morphology	Left fore limb	NS
			Right fore limb	NS
			Left hind limb	NS
			Right hind limb	NS
		Limb grasp	-	NS
		Limb tone	-	NS
		Negative geotaxis	-	NS
		Palpebral closure	-	NS
		Palpebral fissure length	-	NS
		Pinna morphology (left)	-	NS
		Pinna reflex	-	NS
		Positional passivity	-	NS
		Provoked biting	-	NS
		Salivation	-	NS
		Skin color	-	NS
		Startle response	-	NS
		Tail elevation	-	NS
		Tail morphology	-	NS
		Teeth morphology	-	NS
		Toe pinch	-	NS
		Touch escape	-	NS
		Tremor	-	NS
		Trunk curl	-	NS
		Visual placing	-	NS
		Vocalization	-	NS
		Whisker morphology	-	NS
9	Hematology	Percent of basophils (BASO)	-	NS
		Percent of eosinophils (EOS)	-	NS
		Hematocrit (HCT)	-	NS
		Hemoglobin (HGB)	-	NS
		Percent of lymphocytes (LYMP)	-	S
		Mean corpuscular hemoglobin (MCH)	-	NS
		Mean cell hemoglobin concentration (MCHC)	-	NS
		Mean cell volume (MCV)	-	NS
		Percent of monocytes (MONO)	-	S
		Mean platelet volume (MPV)	-	NS
		Percent of neutrophils (NEUT)	-	S
		Platelet count (PLT)	-	NS
		Red blood cell count (RBC)	-	NS
		Red blood cell distribution width (RDW)	-	NS
		Percent of reticulocytes (Retic)	-	NS
		White blood cell count (WBC)	-	NS
		Absolute number of basophils (BASO number)	-	NS
		Absolute number of eosinophils (EOS number)	-	S
		Absolute number of lymphocytes (LYMP number)	-	NS
		Absolute number of monocytes (MONO number)	-	NS
		Absolute number of neutrophils (NEUT number)	-	NS
		Absolute number of reticulocytes (Retic number)	-	NS
10	Urinalysis	Bilirubin	-	NS
		Glucose	-	NS
		Ketone body	-	NS
		Occult blood	-	NS
		Protein	-	NS
		Urobilinogen	-	NS
		pH	-	NS
11	Clinical Blood Chemistry	Albumin (ALB:g/L)	-	NS
		Alkaline phosphatase (ALP)	-	NS
		Alanine aminotransferase (ALT)	-	NS
		Alpha-amylase (AMY)	-	NS
		Aspartate aminotransferase (AST)	-	NS
		Creatine kinase (CPK)	-	NS
		Creatinine (CRE)	-	NS
		Calcium (Ca)	-	NS
		Iron (Fe:mg/dL)	-	NS
		Glucose (Glu)	-	NS
		HDL-cholesterol (HDL-C)	-	NS
		Glycosilated hemoglobin A1c (HbA1c)	-	NS
		Phosphate (IP)	-	NS
		Lactate dehydrogenase (LDH)	-	NS
		LDL-cholesterol (LDL-C)	-	S
		Triglycerides (TG)	-	NS
		Total protein (TP:g/L)	-	NS
		Total bilirubin (T-BIL)	-	NS
		Total cholesterol (T-CHO)	-	NS
		Uric acid (UA:umol/L)	-	NS
		Urea (UN)	-	NS
14	Intraperitoneal glucose tolerance test (IPGTT)	Body Weight	-	S
		Glucose area under the curve (AUC)	-	S
		Blood glucose concentration	-	NS
19	Funduscopy	Optic disc	Color of the optic disc	NS
			Shape of the optic disc	NS
			Size of the optic disc	NS
		Other eye abnormality	Mydriasis after administration of mydriatic drops	NS
			Opacity except in retina	NS
			Visibility of fundus	NS
			Nystagmus	NS
			Deposit except in retina	NS
			Ocular deviation	NS
		Retina	Color of the whole retina	NS
			Visible area of the choroidal vessels	NS
			Appearance of a pigmentation pattern suggesting retinal degeneration in the whole retina	NS
			Appearance of a pattern (not retinal degeneration) on the whole retina	NS
			Appearance of white spots on a portion of the retina	NS
			Hemorrhage	NS
			Other color change in the retina (not white spots or hemorrhage)	NS
			Change around the optic disc	NS
		Vessel	Tortuous pattern of the retinal vessel	NS
			Inconsistent vessel caliber	NS
			Size of the retinal venules	NS
			Size of the retinal arterioles	NS
			Color of the retinal venules	NS
			Color of the retinal arterioles	NS
			Branch pattern of the retinal vessel	NS
22	X-ray	Head	Mandibles	NS
			Maxilla/Pre-maxilla	NS
			Skull shape	NS
			Teeth	NS
			Zygomatic arch	NS
		Trunk	Clavicle	NS
			Fusion of ribs	NS
			Missing cranial rib	NS
			Number of ribs left	NS
			Number of ribs right	NS
			Pelvis	NS
			Scapulae	NS
			Shape of ribcage	NS
			Shape of ribs	NS
		Limb	Brachydactyly	NS
			Digits	NS
			Femur	NS
			Fibula	NS
			Humerus	NS
			Number of digits	NS
			Polydactyly	NS
			Radius	NS
			Syndactyly	NS
			Tibia	NS
			Tibia length	S
			Ulna	NS
		Spine	Kyphosis	NS
			Lordosis	NS
			Scoliosis	NS
			Shape of spine	NS
		Vertebrae: Number	Number of cervical vertebrae	NS
			Number of lumbar vertebrae	NS
			Number of pelvic vertebrae	NS
			Number of thoracic vertebrae	NS
			Transitional vertebrae	NS
		Vertebrae: Shape	Shape of caudal vertebrae	NS
			Shape of cervical vertebrae	S
			Shape of lumbar vertebrae	NS
			Shape of sacral vertebrae	NS
			Shape of thoracic vertebrae	NS
			Shape of vertebrae	S
		Vertebrae: Fusion	Fusion of caudal vertebrae	NS
			Fusion of cervical vertebrae	NS
			Fusion of lumbar vertebrae	NS
			Fusion of sacral vertebrae	NS
			Fusion of thoracic vertebrae	NS
		Vertebral processes: Shape	Caudal processes	NS
			Cervical processes	NS
			Lumbar processes	NS
			Processes on vertebrae	NS
			Sacral processes	NS
			Thoracic processes	NS
		Vertebral processes: Fusion	Fusion of caudal processes	NS
			Fusion of cervical processes	NS
			Fusion of lumbar processes	NS
			Fusion of sacral processes	NS
			Fusion of thoracic processes	NS
		Joints		NS
22	Body Composition (DEXA lean/fat)	Bone Mineral Content (excluding skull)		S
		BMC/Body weight		NS
		Bone Mineral Density (excluding skull)		S
		Body weight		S
		Body fat percentage		S
		Body length		S
		Bone Area (BMC/BMD)		NS
		Fat/Body weight		S
		Fat mass		NS
		Lean/Body weight		NS
		Lean mass		S
24	Electrocardiogram (ECG)	Coefficient of variation (CV)		S
		Heart rate (HR)		S
		JT interval		NS
		PR interval		NS
		QRS interval		NS
		QT interval		NS
		RR interval		S
		Mean R amplitude		NS
		Mean SR amplitude		NS
		Tpeak Tend interval		NS
26	Pathology	Spleen weight to body weight ratio		NS
		Heart weight		NS
		Heart weight to body weight ratio		NS
		Left kidney weight		NS
		Left kidney weight to body weight ratio		NS
		Spleen weight		NS
		Liver weight to body weight ratio		NS
		Right kidney weight		S
		Right kidney weight to body weight ratio		NS
		Liver weight		S
		Body weight		S

Summary of the statistical analyses for the data collected in the PL1 is presented.
All raw data collected and the results of the statistical analyses conducted in this study are deposited in our database, Pheno-Pub (https://ja.brc.riken.jp/lab/jmc/mouse_clinic/m-strain/phenopub_top.html).
Abbreviations: S, statistically significant; NS, not statistically significant.
